# A Case Report of Late Onset Mania Caused by Hyponatremia in a Patient With Empty Sella Syndrome

**DOI:** 10.1097/MD.0000000000002629

**Published:** 2016-02-12

**Authors:** Chung-Hao Yang, Yu-Chen Lin, Po-Han Chou, Hung-Chieh Chen, Chin-Hong Chan

**Affiliations:** From the Department of Psychiatry (C-HY, P-HC); Department of Radiology, Taichung Veterans Hospital, Taichung, Taiwan (H-CC); and Department of Psychiatry, Conde S. Januário General Hospital, Macau, China (C-HC).

## Abstract

Recurrent manic-like episodes can be induced by hyponatremia possibly due to empty sella syndrome. In the present case, the patient was proven to have syndrome of inappropriate antidiuretic hormone (SIADH) secretion with manic symptoms that resolved after the normalization of the plasma sodium level.

To our knowledge, this is the first case of hyponatremia-induced manic symptoms in a patient with empty sella syndrome. More attention should be paid to late-onset mania, because it may be the sign of a more serious medical problem.

## INTRODUCTION

Empty sella syndrome is a condition in which the pituitary fossa appears to be largely empty of tissues and filled by cerebrospinal fluid. Between 20% and 50% of empty sella patients have endocrinologic dysfunction,^1^ and hypopituitarism is one of the most common symptoms. Recurrent hyponatremia may be a presenting feature of empty sella resulting from hypopituitarism and may cause various psychiatric symptoms such as anxiety, depression, mania, and even psychosis. Here, we describe a case of empty sella with recurrent hyponatremia and manic episodes mimicking late-onset bipolar disorder.

## CASE DESCRIPTION

Mrs C is a 69-year-old married and retired Taiwanese woman who lives with her husband. She was referred to our acute psychiatric department because of her manic symptoms including elation and irritability, more talkativeness than usual, decreased need for sleep, and grandiosity for 2 days.

We performed mental status examination to the patient and found that she was oriented and alert, but garrulous with pressured speech, expansive mood, flight of ideas, and delusions of grandeur. On Young Mania Rating Scale, her total score is 24 (scores 4 in elevated mood, 4 in increased motor activity-energy, 1 in sexual interest, 3 in sleep, 2 in irritability, 4 in speech, 1 in language-thought disorder, 2 in content, 0 in disruptive-aggressive behavior, 0 in appearance, and 3 in insight). There is no waxing or waning of cognitive functioning. About the results of the Mini-Mental State Examination, she had intact short-term memory and judgment and no obvious deficit in abstract thinking, and was well oriented and able to perform all activities of daily living without assistance, even with manic symptoms.

She was euvolemic with a plasma sodium level of 116 mmol/L, serum osmolality of 268 mOsm/kg, urine osmolality of 328 mOsm/kg, urine sodium of 62 mmol/L, normal serum creatinine and blood urea nitrogen levels of 0.43 mg/dL and 10 mg/dL, respectively, 7 AM serum cortisol and adrenocorticotropic hormone (ACTH) levels of 12.5 μg/dL and 14.4 μg/dL, respectively, and thyroid stimulating hormone and free T4 blood levels of 1.12 μIU/mL and 9.97 pg/mL, respectively. All characteristics indicated were sufficient to make the diagnosis of syndrome of inappropriate antidiuretic hormone (SIADH) secretion without the need to determine antidiuretic hormone (ADH) levels. She had normal plasma growth hormone and prolactin levels, but below normal plasma luteinizing hormone, progesterone, and follicle stimulating hormone levels. She denied having a history of alcohol and other substance use.

**FIGURE 1:**
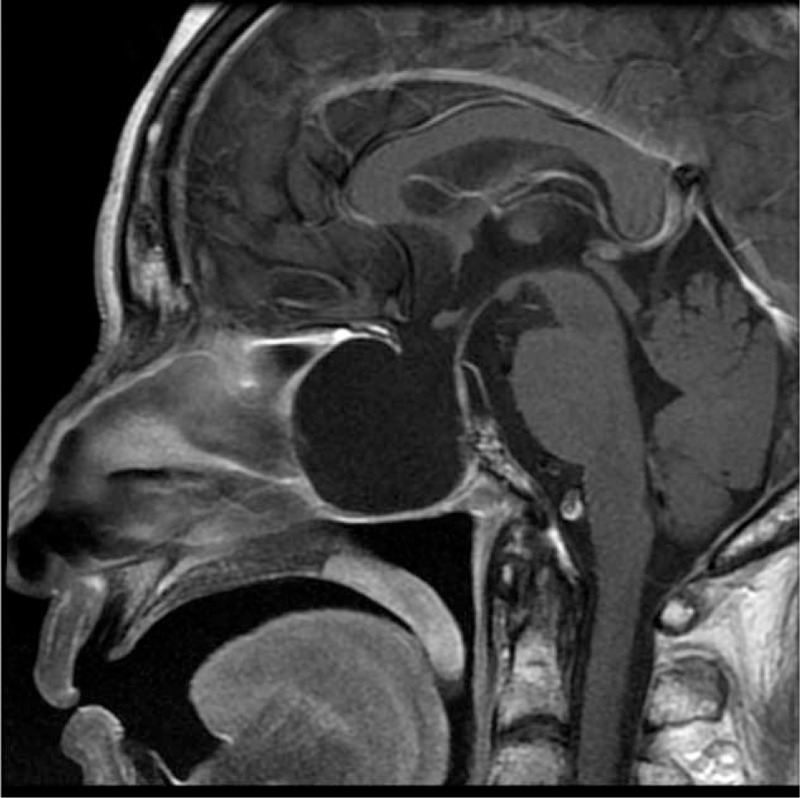
Empty sella combined with intrasphenoidal meningocele formation in magnetic resonance imaging (MRI) scan of brain. Sagittal T1-weighted. Huge cystic structure from pituitary sella herniated into sphenoid sinuses causing sellar floor destruction and wall of sphenoid and ethmoid sinuses remodeling is found.

Brain magnetic resonance imaging revealed an empty sella and intrasphenoidal meningocele formation (Figure 1). Because the patient had never undergone any previous head surgery or radiotherapy, primary empty sella was the preferred diagnosis. Her plasma sodium level had returned to 132 mmol/L by 48 hours after treatment with 300 mL of 3% intravenous NaCl, and her mood became euthymic with no residual mood symptoms. She was then discharged.

This case report does not need ethical approval from ethics committee or institutional review board. The inform consent by the patient is obtained. We already informed the patient about the report during the admission course and after the acute treatment. The case report has been fully explained to the patient and her daughter. They have agreed to participate in this case report.

## DISCUSSION

There have been 3 previously reported cases of hyponatremia induced mania.^2–4^ To our knowledge, this is the first case of hyponatremia-induced manic symptoms in a patient with empty sella syndrome. In this case, the patient was proven to have SIADH, and the manic symptoms resolved after normalization of plasma sodium level.

Hyponatremia is one of the most frequently occurring electrolyte imbalances seen in psychiatric patients. Severe hyponatremia may cause different symptoms that resemble mental illness. According to Ekblom et al,^5^ up to 80% of primary empty sella syndrome patients have some degree of hypothalamic-pituitary dysfunction. Panhypopituitarism, isolated secondary hypogonadism, hyperprolactinemia, and isolated ACTH insufficiency are documented in another case.^6^

Severe hyponatremia due to hypopituitarism and adrenal insufficiency is not a very rare situation. Petridis et al^7^ reported a case of hyponatremia in empty sella caused by hypocortisolism. The most accepted hypothesis is that the decrease in serum cortisol due to panhypopituitarism fails to inhibit expression of ADH, with secondary adrenal insufficiency leading to hyponatremia. However, in our case, the serum cortisol level at 7 AM was 12.5 μg/dL, thus not low enough for the diagnosis of adrenal insufficiency. Nevertheless, stress may elevate basal cortisol level and possibly explain why ADH expression was not inhibited in a manic patient with higher cortisol level.

ADH and ACTH stimulation tests might have helped clarify the relation between empty sella and hyponatremia, but neither test was conducted in the present case. The serum ADH level was not obtained because our laboratory lacked the appropriate kit. However, we had sufficient evidence from clinical features and other laboratory data to make the diagnosis. Steroids may be used to treat low cortisol levels but none was given to our patient because her cortisol level was normal; therefore, insufficient cortisol as a cause of SIADH cannot be ruled out.^8^

Bipolar disorder is a chronic illness with recurring episodes of mania and depression that can last from 1 day to months. The mean age of onset for bipolar I disorder is 18 years.^9^ Yassa et al^10^ selected 50 as the cutoff age for late onset bipolar disorder. About 25% of all bipolar patients are older adults.^11^ In geriatric patients, rather than bipolar disorder, new onset manic symptoms may be due to other medical conditions.^12^ These medical conditions may include multiple sclerosis, stroke, brain tumors, traumatic brain injury, systemic infection with the human immunodeficiency virus, hyperthyroidism, epilepsy, vitamin deficiencies, dementia, and medication adverse effect. Our laboratory examination, brain magnetic resonance imaging survey, history taking, and physical examination excluded most of the possible causes mentioned above. Human immunodeficiency virus testing, electroencephalography, and further dementia survey were warranted to rule out other etiologies, although she had no recent history of sexual exposure, seizures, or dementia.

## CONCLUSION

This case provides evidence for hyponatremia as a possible cause of recurrent manic-like episodes. In our case, the hyponatremia was possibly caused by empty sella. Attention should be paid to late-onset manic symptoms, which might indicate the presence of secondary organic etiologies. Further study of the relationship between empty sella and hyponatremia-induced mania is warranted.
